# The Old in the New

**Published:** 1888-02-11

**Authors:** J. W. Gilbart-Smith


### THE OLD IN THE NEW.

Earth's ripeners, gathered 'neath the reaper's blade,
Gleaned for glad granaries, rich in golden hue,
There part abides, and part within the glade
Awaits the spring to summon it from shade,—
The resurrection of old life in new.

Nor is this truth beyond our reason's range—
The risen life is no unwonted thing,
Germs burst to growth by means of this death-change;
If the dead rose not it were passing strange,
Since even Nature rises with the spring.

—*J. W. Gilbart-Smith.*

